# Design of Computer-Aided Translation System Based on Naive Bayesian Algorithm

**DOI:** 10.1155/2022/1348991

**Published:** 2022-09-06

**Authors:** Zhiqiang Li, Juning Huang, Weixuan Zhong

**Affiliations:** ^1^Foreign Language Department, Ganzhou Teachers College, Ganzhou, Jiangxi 341000, China; ^2^School of Foreign Language, Hechi University, Yizhou, Hechi 546300, China

## Abstract

With the progress of society and the rapid development of science and technology, computer translation technology has become an important auxiliary tool in the fields of software localization and technical translation. This realistic demand has prompted translators to pay more attention to computer translation and have made some useful explorations on this basis. This paper aims to study and discuss computer-aided translation systems based on the fusion of naive Bayesian algorithms. This paper theoretically analyzes some key technologies in computer-aided translation. Computer-aided translation refers to helping translators to translate texts with a series of tools and then proposes a Bayesian classification algorithm. Translation memory technology can solve many practical problems, especially in the machinery manufacturing industry, processing some sentences in documents, which can reduce repetitive labor, unify vocabulary, and make translation styles more coordinated. The experimental results of this paper show that applying the naive Bayes method to the computer-aided translation system can better classify the documents in the translation system, thereby improving the ability of computer-aided translation. When the proportion of professional terms in the article reaches 85%, computer-aided translation has an auxiliary role for the translator. When the proportion of professional terms in the article reaches about 95%, computer-assisted translation can efficiently speed up the work speed and quality of translators. Due to the prosperity of computer translation systems, the duplication of labor for translators has been significantly reduced, and this ensures the consistency of terminology and translation style, so that the fruits of labor are fully utilized.

## 1. Introduction

Machine translation is one of the earliest research topics in natural language processing. It is a comprehensive science that integrates linguistics, mathematics, psychology, and computer science. The current machine translation can no longer meet the actual translation needs, and the advent of the “big data” era has caused many scholars to turn their attention to computer-assisted translation. Computer-aided translation is the use of computer to translate information, which is only an auxiliary tool. Users can decide their own choices according to their own choices. At the same time, the instance corpus can also classify it according to a specific field, improve its translation effect, and make it more in line with the needs of users. This advantage, especially in the case of regional characteristics, a large amount of professional vocabulary, or specific translation, becomes particularly obvious.

Compared with machine translation, computer-assisted translation is more practical and widely used. Regarding how it works, the human translator's translation will be remembered by the software (translation memory), allowing the translator to automatically compare translations with previous translations when they encounter the same translation problem in the future. In addition, translators can also normalize the vocabulary in the document by creating a vocabulary library, thereby guaranteeing the vocabulary in their texts. This paper studies and analyzes the computer-aided translation system based on the fusion of naive Bayesian algorithm and constructs a computer-aided translation system model, aiming to make a certain contribution to teaching translation.

According to the research progress in foreign countries, different researchers have carried out corresponding cooperative research in computer-assisted translation. Yao focused on the current state of the adoption of CAT technology in translation teaching and conducted a survey from the perspective of teachers to investigate people's understanding of it and the problems of its use in translation teaching [1–3]. Wu proposed a semiautomatic evaluation method for machine translation system based on fuzzy mathematics. Firstly, the characteristics of multimedia CAT software are discussed, its working principle is described, its advantages and disadvantages are analyzed and improved, and an optimal solution for translation teaching is given [4, 5]. Drawing on a broad knowledge of the challenges faced in the classroom by students learning English as an additional language and their teachers, as well as the English required for assessment, Excell D examined computer-assisted translation tools and outlined some of the problems and shortcomings of these tools [6]. Zengqiang first introduced the basic content of computer translation methods and then selected some CAT methods to study and respectively introduced the characteristics of translation methods and their application ideas [7]. Dinesh first introduced the concept of parallel corpus, introduced the development of computer translation software, and made a comparative analysis of self-designed on this basis [8]. In order to improve the accuracy of English translation, reduce the error rate of translation results, and improve the accuracy of translation, Nanda and Sari proposed a business English translation architecture design based on speech recognition and wireless communication [9–11]. However, these scholars lack technical proof on the research of computer-aided translation. After research, it is found that the research on computer-aided translation based on naive Bayesian algorithm has certain reliability. In this regard, we have consulted the relevant literature on naive Bayesian algorithm.

At present, some scholars have conducted in-depth research on the naive Bayesian algorithm. Al-batah et al. proposed a local naive Bayesian algorithm based on mutual information and conducted simulation experiments on 5 datasets belonging to different fields, and 9 metrics are used to compare. Numerical simulation results verified the effectiveness of the proposed algorithm for improving link prediction performance [12]. The purpose of Li is to implement an innovative fake news detection method on a recent machine learning classifier to predict fake political news by testing the performance and accuracy of algorithms on fake political news detection and other issues. The comparison results show that the decision tree algorithm has better performance than the naive Bayes algorithm [13]. Personality disorder is a disorder in itself, which can be seen in behavior, mentality, attitude, and so forth, and it brings difficulties to life. Based on this problem, Kusuma et al. used the method of naive Bayes classifier as an early detection of human personality disorder [14, 15]. In order to protect data privacy, Carter and Atkinson proposed a new transition group probabilistic Bayesian algorithm TrGNB. TrGNB is suitable for scenarios where the source domain contains a large amount of labeled data, while the target domain has only a small amount of probability information for unlabeled datasets [16]. However, these scholars did not integrate the naive Bayes algorithm to design and analyze the computer-aided translation system, but only discussed its significance unilaterally. This paper aims to study and discuss computer-aided translation systems based on the fusion of naive Bayesian algorithms.

Computer-aided translation mainly relies on the creation and efficient use of high-quality, large-scale corpora, the use and processing of intermediate methods, and the performance of similarity calculations. It avoids the dilemma of related linguistics well and combines the good situation of the rise of the Internet and the rapid growth of data volume. In this paper, a computer-aided translation system is developed and designed based on the fusion of naive Bayes algorithm, and when we use computers to translate, we can achieve 98% accuracy. However, assisted translation does not reduce the difficulty of translation; it can only provide some references, especially new sentences. More is to let users do it by themselves. In other words, the innovative part is done manually, and the repetitive work is handed over to the computer, thus forming a translation mode of human-computer interaction.

The innovations of this paper are as follows: (1) some key technologies in computer-aided translation are analyzed; (2) Bayesian classification algorithm is proposed; (3) the development and design of computer-aided translation system are experimentally explored.

## 2. Design Method of Computer-Aided Translation System

### 2.1. Key Technologies of Computer-Assisted Translation Based on Translation Memory

**Figure 1:**
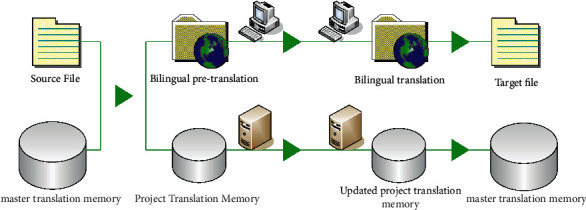
Program translation using computer aids.

Computer-assisted translation is very different from machine translation, which aims to allow machines to completely replace human work. Computer-aided translation realizes translation through a series of tools.  Figure 1 shows the process of program translation using computer-aided assistance. There is a popular saying, “Who will do the real translation work.” Therefore, there are big differences between the two in terms of design ideas and technical support [17, 18]. Memory and terminology are the core technologies of computer-assisted translation. When translators encounter similar translations, the system will match the translations based on previous translations. In the same project, the translator can also establish a vocabulary database to standardize the translation items to ensure the consistency of the translation vocabulary in the same project [19, 20].

**Figure 2:**
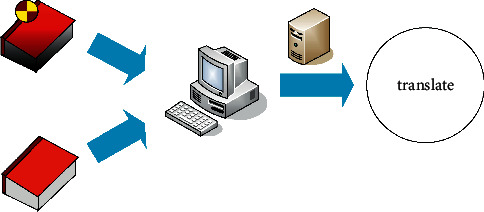
Computer processing knowledge.

Translation memory technology can solve many practical problems, especially in the machinery manufacturing industry; processing some sentences in documents can reduce repetitive labor, unify vocabulary, and make translation styles more coordinated. However, this goal has not yet been achieved in mechanical processing, and some key technologies need to be further studied. In fact, there are three aspects of knowledge processing using computer: acquiring knowledge, expressing knowledge, and using knowledge, as shown in Figure 2. Then, several key technologies are discussed; that is, for the above three problems, corresponding solutions are given, so as to minimize the bottleneck of translation, improve the speed of translation, and improve the quality of translation.

### 2.2. A Naive Bayesian Document Classification Approach in Computer-Aided Translation Systems

A document in a computer-aided translation system is usually a string, and before it can be trained or classified, it must first be represented by a vector space model into a form that is tractable by the learning algorithm. The Bayes theorem is the most valuable scientific research method at present, and it has been widely used in statistics, economics, data mining, artificial intelligence, and so on.

The principle of the naive Bayes classifier is to use the prior probability of the category and the data information of the sample to calculate the posterior probability that the unknown text belongs to a certain category. The “posterior distribution” combines newly observed data with known prior hypotheses or treats it as a known information update, thereby obtaining a new model parameter distribution. The task of Bayesian text classification is to classify the text *t*_*n*_(*u*_1_, *u*_2_, ⋯, *u*_*v*_) to be classified as a vector into the most closely related category *S*(*S*_1_, *S*_2_, ⋯*S*_*m*_), (*u*_1_, *u*_2_, ⋯, *u*_*v*_) is the feature vector of the text to be classified, and *S*(*S*_1_, *S*_2_, ⋯*S*_*m*_) is a given category set. The task of classification is to solve the probability value (*K*_1_, *K*_2_, ⋯*K*_*m*_) that vector *t*_*n*_(*u*_1_, *u*_2_, ⋯, *u*_*v*_) belongs to a given category (*S*_1_, *S*_2_, ⋯*S*_*m*_), where *K*_*m*_ is the probability that *t*_*n*_(*u*_1_, *u*_2_, ⋯, *u*_*v*_) belongs to *S*_*m*_; then, the category corresponding to *max*(*K*_1_, *K*_2_, ⋯*K*_*m*_) is the category to which the text *t*_*n*_(*u*_1_, *u*_2_, ⋯, *u*_*v*_) belongs. Therefore, the text classification problem in computer-aided translation systems is described as solving the maximum of the following equation:

(1) $$\begin{array}{c} K\left(s_{m}|t_{n}\right)=\frac{K\left(t_{n}|s_{m}\right)K\left(s_{m}\right)}{K\left(t_{n}\right)}. \end{array}$$

Here, for all given categories, the denominator is a fixed value of *K*(*t*_*n*_), so solving the maximum value of the above formula is converted to solving the maximum value of the following formula:

(2) $$\begin{array}{c} K\left(s_{m}|t_{n}\right)\infty K\left(t_{n}|s_{m}\right)K\left(s_{m}\right). \end{array}$$

Naive Bayes classifier is “naive”; that is, the text feature vector attribute *u*_1_, *u*_2_, ⋯, *u*_*v*_ in the translation system is independent and identically distributed, and its joint probability distribution is equal to the product of the probability distribution of each attribute feature. Using this assumption, we have

(3) $$\begin{array}{c} K\left(t_{n}|s_{m}\right)=\prod_{h=1}^{v}K\left(u_{nh}|s_{m}\right). \end{array}$$

*K*(*u*_*nh*_*|s*_*m*_) represents the conditional probability of word *u*_*h*_ in class *S*_*m*_, that is, the contribution value (or weight) of word *u*_*h*_ to document *t*_*n*_ belonging to class *S*_*m*_. The eigenvalues of the text in the translation system are calculated, and each eigenvalue is assigned a weight value. The size of the weight value represents the importance of the text feature, the feature with the largest weight value is the decisive feature, and the decisive feature can represent a certain type of weight to represent the importance of the feature. Generally speaking, the larger the weight is, the more the feature can represent a certain class.

The purpose of text classification in computer-aided translation systems is to find the most likely category of a document, and the most likely category of a document in naive Bayesian text classification is the category *S*_*map*_, where the maximum a posteriori probability (MAP) is located. *S*_*map*_ of document *t*_*n*_ belonging to class *S*_*m*_ can be expressed as the following formula, where *K*(*s*_*m*_) represents the prior probability that a document *t*_*n*_(*u*_1_, *u*_2_, ⋯, *u*_*v*_) belongs to class *S*_*m*_.

(4) $$\begin{array}{c} S_{\text{map}}=\arg\max_{S_{m}\in S}K\left(s_{m}|t_{n}\right)=\arg\max_{S_{m}\in S}K\left(s_{m}\right)\prod_{h=1}^{v}K\left(u_{nh}|s_{m}\right). \end{array}$$

This formula is the solved classification function for text classification. But in the actual calculation, because the formula contains a large number of conditional probabilities multiplied together, the floating-point result underflow may be caused. To solve this problem by taking the logarithm of both sides of the equation instead of multiplying the probabilities, the expression for calculating *S*_*map*_ can be expressed as

(5) $$\begin{array}{c} S_{\text{map}}=\arg\max_{S_{m}\in S}K\left(s_{m}|t_{n}\right)=\arg\max_{S_{m}\in S}\left[\log\;K\left(s_{m}\right)+\sum_{h=1}^{v}\log\;K\left(u_{nh}|s_{m}\right)\right]. \end{array}$$

In order to complete the calculation of this classification function, it is necessary to complete the calculation of two main parameters, namely, the calculation of the class prior probability *K*(*s*_*m*_) and the conditional probability *K*(*u*_*nh*_*|s*_*m*_) of the characteristic word class. The polynomial model is used for the calculation, and the calculation method of these two parameters is as follows.

For the calculation of class prior probability *K*(*s*_*m*_), it is easy to obtain based on the proportion of each class in the training data; that is, the prior probability calculation expression of class *S*_*m*_ is as follows:

(6) $$\begin{array}{c} K\left(s_{m}\right)=\frac{P\left(s_{m}\right)}{\sum_{h}P\left(sh\right)}. \end{array}$$

*P*(*s*_*m*_) is the number of sample documents contained in class *s*_*m*_, and ∑_*h*_*P*(*sh*) is the total number of all sample documents.

For the estimation of the conditional probability of the feature word, and to avoid the estimated value being zero, the Laplace smoothing technique is used to simply add one to each item, and the following formula is used for calculation in the study:

(7) $$\begin{array}{c} K\left(u_{nh}|s_{m}\right)=\frac{P_{nm}+1}{\sum_{h}P_{hm}+\left|L\right|}. \end{array}$$

*P*_*nm*_ is the normalized weight of feature word *u*_*n*_ in class *s*_*m*_ documents, ∑_*h*_*P*_*hm*_ is the sum of the normalized weights of all feature words in class *s*_*m*_, and |*L*| is the smoothing factor. Its value is the number of feature word types that appear in all sample documents in the computer-aided translation system.

## 3. Experimental Results of the Design of a Computer-Aided Translation System

### 3.1. Overall Framework of the Computer-Aided Translation System

The system is based on a client/server architecture. Since this translation system needs to be embedded in CAPP, in general, batch translation should be realized. The translation process does not require manual intervention. After the batch translation is completed, it is modified on the interface, so a dynamic link library version is implemented, which is directly called by CAPP. The core of this translation system adopts translation memory technology. When it was just put into use, the sentences that could be translated were limited and had to be translated through human-computer interaction. Therefore, a version with an interface was developed, which mainly used the version with an interface to explain related issues.

**Figure 3:**
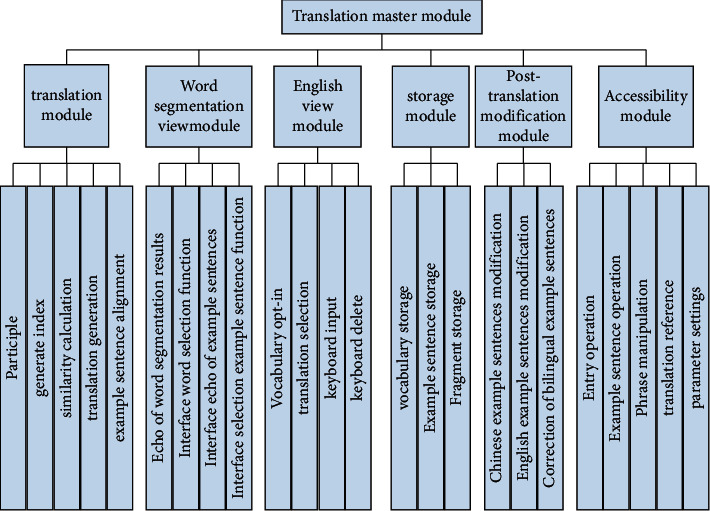
Overall function diagram of the system.

The overall function diagram of the system is shown in Figure 3.

From the needs of the actual system design, this paper realizes the overall flow of the program through the following steps:

1. Input the Chinese sentence to be translated, look up the bilingual dictionary, and divide the sentence to be translated into various words.
2. Four overall similarity indexes are generated according to the requirements of overall similarity calculation for the segmented vocabulary, which are used for preliminary retrieval of similar example sentences in the example sentence database.
3. Use the index generated in the second step to search for similar Chinese example sentences that meet the search conditions on the overall structure in the example sentence library, and then use the fine-tuning similarity to select the best example sentences.
4. Extract the lexical alignment relationship between Chinese example sentences and example sentences translations, and combine the results of the similarity to complete the lexical alignment between the sentences to be translated and the example sentences translations.
5. Using the alignment result, on the basis of the sentence translation of the example sentence, use certain conversion rules to generate the translation we need.
6. When the user feels that the generated translation does not meet the requirements, he can manually modify the result and save the result.

**Figure 4:**
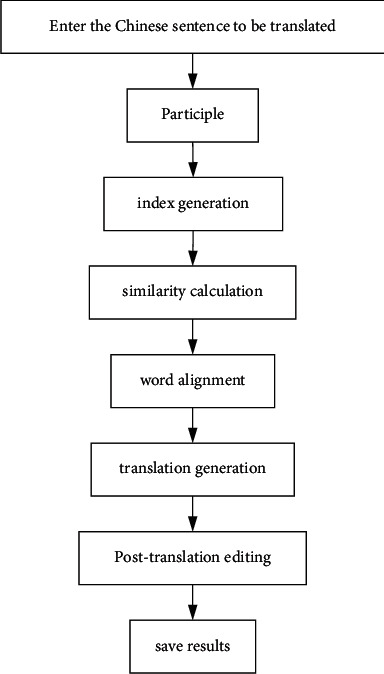
Overall system flow.

The overall flow chart is shown in Figure 4.

#### 3.1.1. Word Segmentation Module

In view of the advantages and disadvantages of various word segmentation algorithms, as well as the specific research problems and word segmentation goals, this paper selects the algorithm of word segmentation using forward maximal matching method and setting up segmentation mark method for initial segmentation of words.

The basic idea of the maximum forward maximum matching method is to cut out the longest word from left to right in the input sequence. First, some segmentation marks were established, mainly including various punctuation marks, English characters, Arabic numerals, etc. When performing word segmentation, the entire sentence to be translated is scanned, and these segmentation marks are searched. According to these marks, the sentence is divided into several shorter segments. Then, according to the determination of a maximum possible word length *k*, this article takes *k* words in the current string sequence in the segment and searches in the bilingual dictionary. If no word of this length is found, *k* is reduced by 1, and the search continues and so on, until the word is found and segmented. If it is not found until *k* = 1, get a single word. This method is simple to implement but has a certain miscut rate, and its miscut rate is 1/169. According to the ambiguity flag in the bilingual dictionary, the value of *k* can be reduced by 1 to continue the segmentation.

If there is a new word in the result of word segmentation, its part of speech, part-of-speech subclass, and unique number cannot be known, and the subsequent similarity calculation and translation generation cannot be continued. The user only needs to input the new words into the bilingual dictionary database on the interface and then perform word segmentation again to solve the problem.

**Figure 5:**
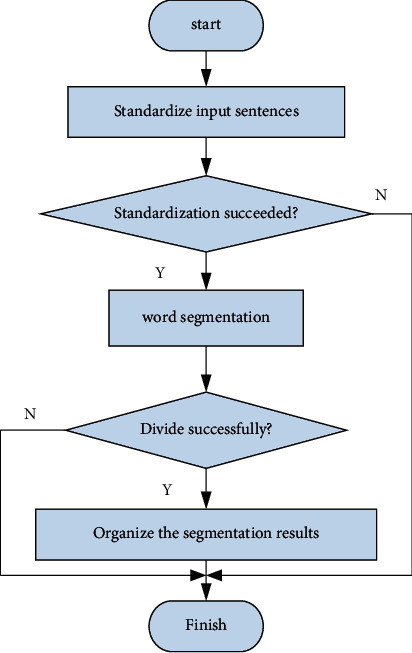
Word segmentation flow chart.

The ultimate goal of segmentation is to segment the sentence into a form that is believed to be correct. The specific process is shown in Figure 5.

The subblock library mainly stores word fragments composed of more than two non-keywords. Since the subblock library stores all the fragments composed of non-keywords, the form of the structure string index is not involved. If two sentences are to be determined to be similar by comparing word fragments with each other, it has been shown that the two sentences are different to a large extent. From the consideration of similarity theory and practical application, the form of part-of-speech string index is also not required. The subblock library structure design is shown in Table 1.

#### 3.1.2. Index Generation Module

Word segmentation is only the first step. The purpose is to search for similar or identical example sentences in the example sentence database, so as to generate translations that meet the requirements. The task to be completed by the index generation module is to generate unambiguous indexes of overall similarity on the basis of the word segmentation results.

According to the four overall similarities, four overall similarity strings are generated, and they are used to retrieve sentences similar to the sentences to be translated in the sentence database. The four overall similarity strings are referred to as the power string index, the part-of-speech subclass string index, the part-of-speech string index, and the structure string index, respectively.

When generating the ID string index, it is sufficient to connect the order of each word of the sentence to be translated that has been divided into words.

When generating the part-of-speech subclass string index, the part of speech and part-of-speech subclasses of each word of the sentence to be translated that have been divided into words can be connected in an order as a whole.

When generating a part-of-speech index, it is necessary to distinguish between keywords and non-keywords. For keywords, the part of speech and part-of-speech subclasses are still treated as a whole, while for non-keywords, only the part-of-speech part is taken. The part-of-speech subclass is not limited, and the number “0” is used instead. Then, the non-keyword part of speech and part-of-speech subcategories are also treated as a whole, and then the words can be connected according to the order in which the words appear in the sentence to be translated.

When generating the structure string index, special processing should also be done, and the keywords and word fragments should be processed separately. Similar to generating part-of-speech string indexing, keywords treat part of speech and part-of-speech subclasses as a whole, and word fragments are also treated as a whole. The part of speech and part-of-speech subclasses of the word fragments are all replaced with “0,” and then, the keywords and the word fragments are sequentially connected to form the structure string index.

According to the grammatical semantic item principle of the word segmentation specification when building a bilingual dictionary, it can be known that the same word may have more than one entry in the bilingual dictionary due to different parts of speech or subclasses of parts of speech. For example, the word “processing” has two parts of speech: verb and noun.

When there is ambiguity between part of speech and part-of-speech subcategories, it is impossible for a computer to identify which part of speech or semantics to use in the sentence. In this translation system, the computer-aided translation principle is adopted, and the tasks that cannot be completed by the machine are handed over to the user to complete some tasks that only humans can complete. However, the machine can do some automatic matching work, inferring the choice of the ambiguity according to the degree of similarity. If the ambiguity appears in a sentence, the module for generating the index will generate the corresponding index for the word according to the number of ambiguities, a part of speech or a subclass of part of speech that corresponds to the occurrence of ambiguity for that word in each index.

The appearance of this ambiguity makes it difficult to match the segmentation results and indexes of words. In order to correspond to each index, the segmentation results of the corresponding part of speech and part-of-speech subclasses must be given at the same time when the index is generated to left to select the most suitable form after calculating the similarity.

Regarding similarity, alignment, and translation generation modules, the implementation of this system preliminarily retrieves similar Chinese example sentences through the index generated by the sentences to be translated into the example database and then finds the most similar example sentences according to the fine-tuned similarity and, by the way, completes the alignment of the to-be-translated example sentences and the Chinese example sentences. The translation of the example sentence can be used as a template, and a certain quality of translation can be obtained by modifying it. Although similarity, alignment, and translation generation are different in principle, they can be organically combined in system implementation, which saves a lot of computation and reduces the complexity of program design. The number, part of speech, part-of-speech subcategory, and other information of each word in the bilingual dictionary for the segmented Chinese example sentences are all implied in the four index structures, and they can be extracted in sequence when they are used. The structure table of the example database is shown in Tables 2 and 3.

Querying similar instances from the instance database can be realized according to the similarity between the translated sentence and the instance. In four aspects, the overall similarity between the translated sentence and the instance has only four levels, namely, the overall similarity of ID strings, the overall similarity of part-of-speech subclasses, the overall similarity of part-of-speech subclasses, and the overall similarity of structural strings and the overall similarity. If an example sentence is retrieved from the four overall similarities, it means that at least one of the four overall similarities is 100% similar. A similarity percentage of 0% means that there are no instances in the memory that are similar to the sentence to be translated. Between 0% and 100%, it indicates that the sentence to be translated has some similarity with the instance in the memory bank. The details are shown in Table 4.

When similar example sentences are retrieved, the best similar example sentences should be selected. At this time, four fine-tuning similarities are implemented, that is, the similarity degree of ID string fine-tuning, the fine-tuning similarity of part-of-speech subclass strings, the fine-tuning similarity of part-of-speech strings, and the fine-tuning similarity of structure strings. When fine-tuning the similarity calculation, the alignment of the sentence to be translated and the Chinese example sentence is completed by the way, and the words that are inconsistent between the Chinese example sentence and the sentence to be translated are directly replaced to complete the translation generation.

#### 3.1.3. Posttranslation Processing

The above word segmentation, index generation, similarity calculation, alignment technology, and translation generation are all automatically completed by the translation system according to certain rules. Of course, we cannot completely hand over the translation work to the machine. As far as the quality of the current translation software is concerned, it has not yet reached a satisfactory level. The translation system completely translates the sentences to be translated according to the user's previous translation method, and then the user revises the translation until the user is satisfied.

The user can complete the modification of the translation through the modification and saving interface of the translation result. If the user is satisfied with the revised final translation result, the final translation result can be saved for future translation through the “Save” button shown on the interface.

All of the above is done under the premise that similar example sentences can be found. If there is no example sentence similar to the sentence to be translated, the translation of the new sentence must be completed manually after the word segmentation. The user translates as needed with the help of the segmentation results given by the computer, that is, a human-machine-assisted translation method.

### 3.2. Deconstruction of Experimental Results

**Figure 6:**
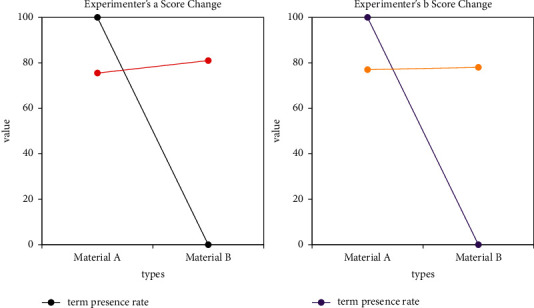
Score changes for translators a and b.

**Figure 7:**
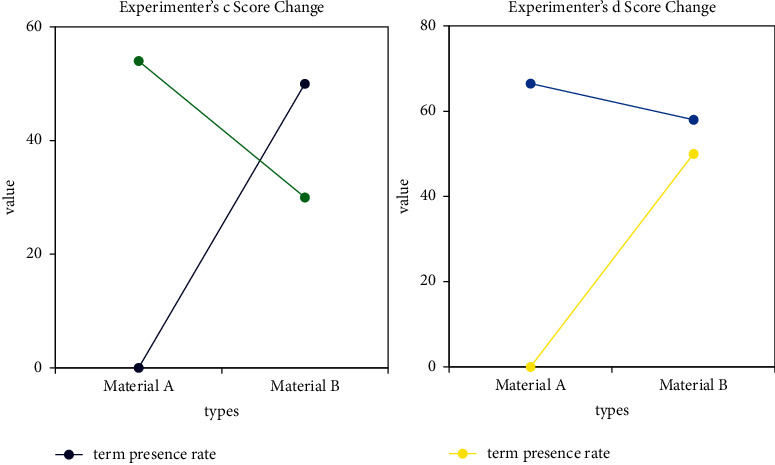
Score changes for translators c and d.

Bayesian algorithm is based on Bayesian theory and uses the knowledge of probability and statistics to classify sample data. The Bayesian classification method has a very low rate of misjudgment due to its solid mathematical foundation. The Bayesian algorithm has the characteristics of combining the prior probability and the posterior probability, which not only avoids the subjective bias caused by only using the prior probability, but also effectively overcomes the overfitting caused by the information of a single sample. In this regard, this paper applies the naive Bayes method to the computer-aided translation system, which can better classify the documents in the translation system. In the translation process, we usually use a special list of professional terms. When translating material A, the experiment was set to no computer assistance. When doing material B, the experiment set the term existence rate to 50%. The performance map of each experimental object in different variables in the two experiments before and after is made, as shown in Figures 6 and 7.

As can be seen from the figure, in the translation of the two materials, the translation quality of most translators improved as the term presence rate decreased, and it increased as the term presence rate decreased.

The experimental results show that when the article term existence rate is 85%, computer assistance has helped translators. When the article term existence rate reaches about 95%, computer assistance effectively helps translators improve the time efficiency of translation.

**Figure 8:**
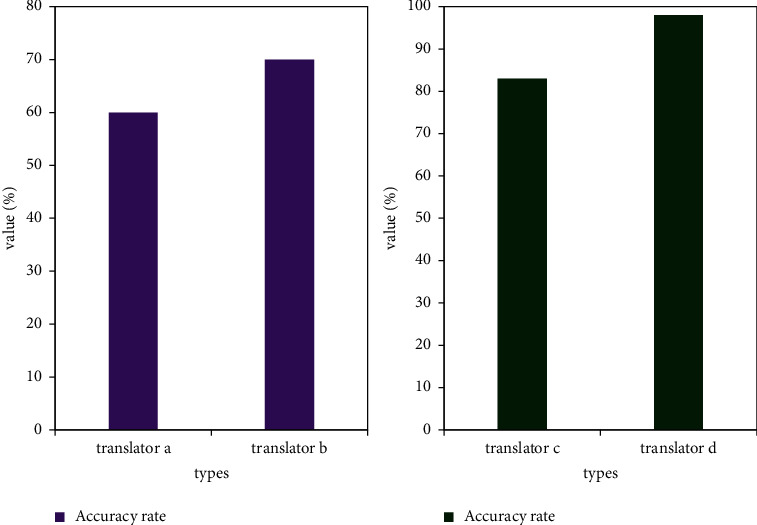
Comparison of computer-aided translation effects.

Computer-assisted translation systems can automatically scan jobs and identify mistranslated words, simple misspellings, or incorrect punctuation.  Figure 8 shows the accuracy of the translator's translation of the article and the use of computer-assisted translation.

In the figure, translators a and b are purely manual translations, translator c is purely computer translation, and translator d uses a computer to assist himself in translation. In these applications, computer translation can achieve 98% accuracy.

## 4. Conclusion

Naive Bayesian method has played a great role in character recognition and image recognition. It uses existing classification rules to classify unknown text or pictures, thereby realizing the classification of a specific text or picture. This paper aims to study and discuss computer-aided translation systems based on the fusion of naive Bayesian algorithms. From the above research content and system implementation, the emergence of computer-aided translation systems can significantly reduce the repetitive work of translators and ensure the unity of terminology and translation modes and styles, which enables the sharing and reuse of labor results. But there is one thing that must be noted; that is, the auxiliary translation system does not reduce the difficulty of translation in essence, but only provides translation references, including segmentation results reference, example sentence reference, and translation generation reference. Especially for the translation of new sentences, most of the work is done by the user; that is to say, the innovative part is done by people, and the repetitive work is done by the computer, thus realizing the translation method of human-computer mutual assistance. However, due to the limitations of time and technology, the specific application of the naive Bayes algorithm in the computer-aided translation system has not been carried out in detail, and we will further carry out experiments to discuss this in the future. However, this paper does not consider the choice of words during translation generation and does not solve the theory and implementation of the singular and plural of English words and part-of-speech transformation, which is of great significance to the improvement of translation quality.

## Figures and Tables

**Table 1:** Subblock library structure table.

Serial number	Appellation	Illustration
1	Chinese snippet	
2	Segmentation form of Chinese fragment	Segmentation results of Chinese fragments
3	Fragment translation	
4	Segmentation of fragmented translations	Including the alignment relationship between Chinese and English
5	ID string index	
6	Part-of-speech subclass string index	
7	Result index	Indicating the central meaning of the fragment

**Table 2:** Example sentence library structure Table 1.

Serial number	Appellation	Illustration
1	Chinese example sentences	—
2	Split form of Chinese example sentences	—
3	Example translation	—
4	The split form of the translation of the example sentence	Including the alignment relationship between Chinese and English

**Table 3:** Example sentence library structure Table 2.

Serial number	Appellation	Illustration
5	ID string index	—
6	Part-of-speech subclass string index	—
7	Part-of-speech string index	—
8	Structure class string index	—
9	Adder name	Adder of recording language

**Table 4:** Match categories.

Similar percentage	Matching degree
0%	Completely mismatched
0%∼100%	Fuzzy match
100%	Exact match

## Data Availability

The data used to support the findings of this study can be obtained from the corresponding author upon request.
